# Prevalence and predictors of high preoperative anxiety among adults undergoing elective surgery in Eastern Nepal: a multicenter cross-sectional study

**DOI:** 10.1097/MS9.0000000000005248

**Published:** 2026-06-15

**Authors:** Sujan Dhakal, Muskaan Shrestha, Elija Gautam, Khadananda Regmi, Salin Dhakal, Sanjib Kumar Sharma

**Affiliations:** a Department of Anaesthesia and Critical Care, Provincial Hospital Bhadrapur, Jhapa, Nepal; b Department of Anaesthesia and Critical Care, Om Sai Pathibhara Hospital, Jhapa, Nepal; c Department of Obstetrics and Gynecology, Provincial Hospital Bhadrapur, Bhadrapur, Nepal; d Department of Anthropology, Mechi Multiple Campus, Jhapa, Nepal; e Department of Anaesthesia, National Medical College, Birgunj, Nepal; f BP Koirala Institute of Health Sciences (BPKIHS), Dharan, Nepal

**Keywords:** Amsterdam Perioperative Anxiety and Information Scale, anesthesia, clinician-rated anxiety scale, cross-sectional studies, elective surgery, Nepal, preoperative anxiety

## Abstract

**Background:**

Preoperative anxiety (PA) is a common yet under-assessed psychological state that affects surgical outcomes and patient well-being. This study aimed to determine the prevalence of high PA and its associated factors in adult patients undergoing elective surgery in Eastern Nepal.

**Methods:**

A multi-center analytical cross-sectional study was conducted at two referral hospitals in eastern Nepal. Patients completed the Amsterdam Perioperative Anxiety and Information Scale (APAIS) in the preoperative holding area, and an attending anesthesiologist provided a clinician-rated anxiety scale (CRS). High anxiety was defined as an APAIS or CRS score ≥11. Demographics and clinical variables were recorded. Descriptive statistics summarized the sample, and the prevalence of high anxiety was calculated with 95% confidence intervals. The chi-square test and multivariable logistic regression were used to identify associations and predictors of high anxiety and categorical variables. Internal reliability (Cronbach’s alpha) and correlation were assessed for both scales.

**Results:**

Of the 512 patients included in the study, the mean age was 42.2 ± 14.8 years, and 59.8% were female. High preoperative anxiety was identified in 11.1% of patients using the APAIS and in 14.3% using the CRS. A high need for information was reported in 6.8% (APAIS) and 7.8% (CRS) of participants. Multivariable analysis revealed that younger age, female sex, gynecological surgery, public hospital setting, previous anesthesia exposure, and high information need were independently associated with higher anxiety. The strongest predictor was high information need (odds ratio = 12.9). APAIS and CRS displayed significant agreement (κ = 0.754), with CRS exhibiting strong diagnostic precision.

**Conclusion:**

In this Eastern Nepal cohort, the prevalence of high preoperative anxiety was significantly lower compared to many reports in other settings. Tailored interventions are needed, especially for young patients and those with higher information requirements. High concordance between patient self-reports and clinician ratings suggests both methods reliably detect high PA.

## Introduction

Preoperative anxiety comprises the spectrum of cognitive apprehensions, emotional distress, and physiological changes that patients experience when anticipating surgery. These responses, driven by hypothalamic–pituitary–adrenal axis stimulation and sympathetic activation^[^1^]^, can complicate perioperative management through altered hemodynamics, increased anesthetic requirements, and delayed recovery^[^2^]^. Beyond immediate physiological impacts, heightened anxiety has been linked to greater postoperative pain, increased opioid consumption, longer hospital stays, and lower patient satisfaction^[^2^]^.

HIGHLIGHTS

- Multicenter assessment of preoperative anxiety in Eastern Nepal (*n* = 512).
- Use of the “gold standard” tool and the clinician’s rating of anxiety.
- Low prevalence [11.1% Amsterdam Perioperative Anxiety and Information Scale (APAIS); 14.3% clinician-rated anxiety scale (CRS)] compared to other low-and middle-income countries.
- High information need (odds ratio 12.9) emerged as the strongest predictor of anxiety.
- Good concordance between patient self-report (APAIS) and clinician-rated scale (CRS).

Preoperative anxiety is a complex and multifaceted response that often starts with patients’ concerns about surgical course or past negative experiences. Its intensity and duration vary, so early recognition and appropriate management are crucial. It often begins with cognitive worry; patients may dwell on worst-case scenarios, complications, or past suboptimal experiences^[^3^]^. Inadequate health literacy might exacerbate these anxieties, particularly when the surgical procedure is not well comprehended. Patients may feel fear, grief, or dissatisfaction, particularly if they perceive surgery as a threat to their personal autonomy. Insufficient social support or previous traumatic encounters with healthcare professionals frequently exacerbate these emotions. Anxiety physiologically triggers the body’s stress reaction, resulting in elevated heart rate, blood pressure, and stress hormone levels, which can disrupt anesthetic control and recovery. The cognitive, emotional, and bodily components are intricately interconnected, frequently amplifying one another in a loop that exacerbates discomfort. Therefore, comprehending this intricate interaction is crucial. Mitigating preoperative anxiety necessitates more than mere reassurance; it demands a systematic, patient-centered strategy that acknowledges individual apprehensions, guarantees transparent communication, and provides supportive care that can enhance surgical outcomes.

A recent systematic review and meta-analysis incorporating over 14 000 patients reported a global pooled prevalence of 48% for high preoperative anxiety [95% confidence interval (CI): 39–47%]^[^4^]^. Prevalence varies by region, reaching upwards of 56% in African low-and middle-income countries (LMICs), 62% in Asian LMICs, and as low as 24% in North America^[^5^]^. In Southeast Asia, studies from India and Pakistan have documented rates ranging from 31% to 97%, illustrating the influence of cultural, socioeconomic, and healthcare system factors on anxiety expression and measurement.

A range of validated instruments, including the Amsterdam Preoperative Anxiety and Information Scale (APAIS), the State-Trait Anxiety Inventory (STAI), and the Hospital Anxiety and Depression Scale (HADS), facilitate standardized anxiety assessments^[^6^]^. The APAIS, as originally developed by Moerman *et al*^[^7^]^, comprises six items split into two subscales: an anxiety scale (items 1, 2, 4, and 5), with two items related to anesthesia and two to surgery, and a need-for-information scale (items 3 and 6). In the initial validation, the anxiety and information subscales demonstrated Cronbach’s alphas of 0.86 and 0.72, respectively^[^7^]^. In the Nepalese adaptation, both subscales retained acceptable internal consistency, with Cronbach’s alpha coefficients of 0.79 for anxiety and 0.77 for information^[^8^]^.

Self-reporting tools, like APAIS, are considered the gold standard for evaluating the internal, subjective experience of anxiety; however, factors such as a patient’s self-awareness, need for social acceptance, or reliance on “blunting” coping strategies can limit their efficacy. The CRS tool can serve as a complementary tool to address these limitations^[^6^]^. In the preoperative meeting, anesthesiologists can use the CRS to determine indicators of anxiety, including behavioral signals, physiological agitation, and verbal cues.

Most validation and prevalence studies remain confined to single centers or high-income settings, limiting their generalizability to diverse cultural and resource environments. In South Asia, for example, single-center reports have documented preoperative anxiety rates ranging from 23% to 52%, but methodological heterogeneity and geographic concentration leave gaps in our understanding of broader LMIC contexts^[^6,9^]^.

In Nepal, single-center studies yield broad estimates, ranging from 7% to 83.9% across different perioperative settings and varying anxiety scales^[^6,8–12^]^. However, small sample sizes, differing assessment tools, and geographic concentration limit these studies. Notably, the easternmost suburban region, characterized by unique sociocultural dynamics and dual public–private referral centers, lacks a comprehensive, multicenter evaluation of preoperative anxiety.

We hypothesize that in adult elective surgical patients from eastern Nepal, the prevalence and determinants of high preoperative anxiety are shaped by demographic factors, clinical history, and psychosocial variables^[^13,14^]^. Furthermore, we propose that both the APAIS and a parallel CRS will demonstrate excellent reliability and diagnostic accuracy in this context.

The primary objective of this study was to determine the prevalence and key predictors of high preoperative anxiety at two referral hospitals in eastern Nepal. Secondary objectives included evaluating the internal consistency and criterion validity of the APAIS and CRS, and identifying independent factors associated with elevated anxiety.

This cross-sectional study has been reported in accordance with the STROCSS guidelines^[^15^]^ and in compliance with the TITAN Guidelines 2025^[^16^]^.

## Materials and methods

### Study design and setting

We conducted an analytical multicenter cross-sectional study over 4 months to assess the prevalence of high preoperative anxiety and its associated factors at two first-referral institutions in Eastern Nepal. These centers, a public, provincial government-run facility and a private multispecialty hospital, were selected to reflect diverse healthcare environments within the same geographic and sociocultural area. Both hospitals serve a similar patient population from the surrounding Terai and Hilly regions and follow identical preoperative counselling protocols. During the pre-anesthetic check-up, anesthesia providers inform patients about the anesthesia technique, fasting instructions, and basic procedural details. This standardized approach ensures consistent patient information delivery and minimizes potential differences in counselling quality.

### Study population

The study included patients aged 18 years or older who were scheduled to undergo elective surgical procedures under anesthesia. A total of 512 patients were enrolled after obtaining written informed consent. Exclusion criteria included refusal to participate, the presence of documented psychiatric illness or intellectual disability, current use of anxiolytics or antidepressants, language barriers, American Society of Anaesthesiologists Physical Status (ASA-PS) class IV or above, and patients undergoing daycare surgeries.

### Sample size and sampling technique

The sample size was calculated using the formula:

$$N=Z^{2}(p\times q)/e^{2}$$

A study conducted by Acharya *et al*^[^6^]^ at a tertiary teaching hospital in central Nepal evaluated 110 adult patients undergoing elective gynecological and gastrointestinal surgeries for elevated anxiety levels in the wards, 1 day prior to surgery, utilizing the APAIS tool, which revealed a prevalence rate of 51.81%. Based on this prevalence (*P*) of 51.81%, a 95% confidence interval (*Z* = 1.96), and a 5% margin of error (*e* = 0.05), the minimum sample size was determined to be 384. Incorporating a 10% buffer for non-responses, the final minimum sample size was established at 422. Adult patients undergoing elective surgery at two hospitals were recruited through convenience sampling.

## Data collection procedure

Eligible patients, who were previously declared fit for anesthesia and had received one round of perioperative counselling, were approached in the preoperative holding area approximately 10–15 minutes before transfer to the operating theater. This timing was chosen to capture the peak of situational anxiety. After obtaining informed written consent, trained anesthesiologists, anesthesia assistants, or perioperative care nurses administered the Nepali version of the APAIS via interview. Responses were recorded on a 5-point Likert scale (1 = not at all, 5 = extremely).

Simultaneously, a different anesthesiologist, blinded to the APAIS score and not involved in the initial counselling, independently rated the patient’s anxiety using the CRS. This tool mirrors the APAIS structure, allowing clinicians to rate perceived patient anxiety and information needs. To minimize assessment bias and enhance consistency, all CRS raters and APAIS interviewers underwent a training session on the structured use of scales after protocol approval. This included calibration using pilot cases, discussion of item anchors, and clarification of scoring criteria.

All procedures for collecting and managing data followed ethical guidelines to ensure participant confidentiality and protect data integrity. Completed questionnaires were securely stored in locked cabinets and subsequently digitized using password-protected systems that were accessible only to authorized personnel.

Scoring Criteria^[^7^]^

**APAIS total anxiety score (SUM C)** = APAIS1 + APAIS2 + APAIS4 + APAIS5

High anxiety is defined as SUM C ≥ 11.

**APAIS information need score (SUM I)** = APAIS3 + APAIS6

High information need is defined as SUM I ≥ 5.

CRS scores were interpreted using the same cut-off values for anxiety and information need as APAIS.

## Data analysis

The data were entered into Microsoft Excel and analyzed using IBM SPSS version 26.0. Data were checked for completeness at the point of entry, and any missing responses were addressed through immediate verification with the source documents. Normality was assessed using the Shapiro–Wilk test. Variables that met normality assumptions were summarized using means and standard deviations, while those that did not were presented as medians with interquartile ranges. Descriptive statistics were used for demographic and clinical characteristics.

Chi-square tests were applied to evaluate associations between high anxiety and variables such as age, gender, marital status, education, comorbidities, surgical specialty, and type of hospital. Logistic regression was performed to identify independent predictors of high anxiety. Covariates for the multivariable logistic regression model were selected based on a *P*-value of <0.05 in the bivariate analysis or identified as clinically relevant factors from existing literature. To address potential confounding, we employed a multivariable regression approach, calculating adjusted odds ratios (AOR) to determine the independent effect of each predictor while controlling for all other included variables. The final model’s goodness-of-fit was assessed using the Hosmer–Lemeshow test. A *P*-value <0.05 was considered statistically significant for all inferential analyses. Correlation coefficients and Cohen’s kappa were used to assess the relationship and agreement between APAIS and CRS scores. Receiver operating characteristic (ROC) analysis was conducted to evaluate the diagnostic accuracy of CRS in identifying high anxiety as classified by APAIS.

## Results

**Figure 1.**
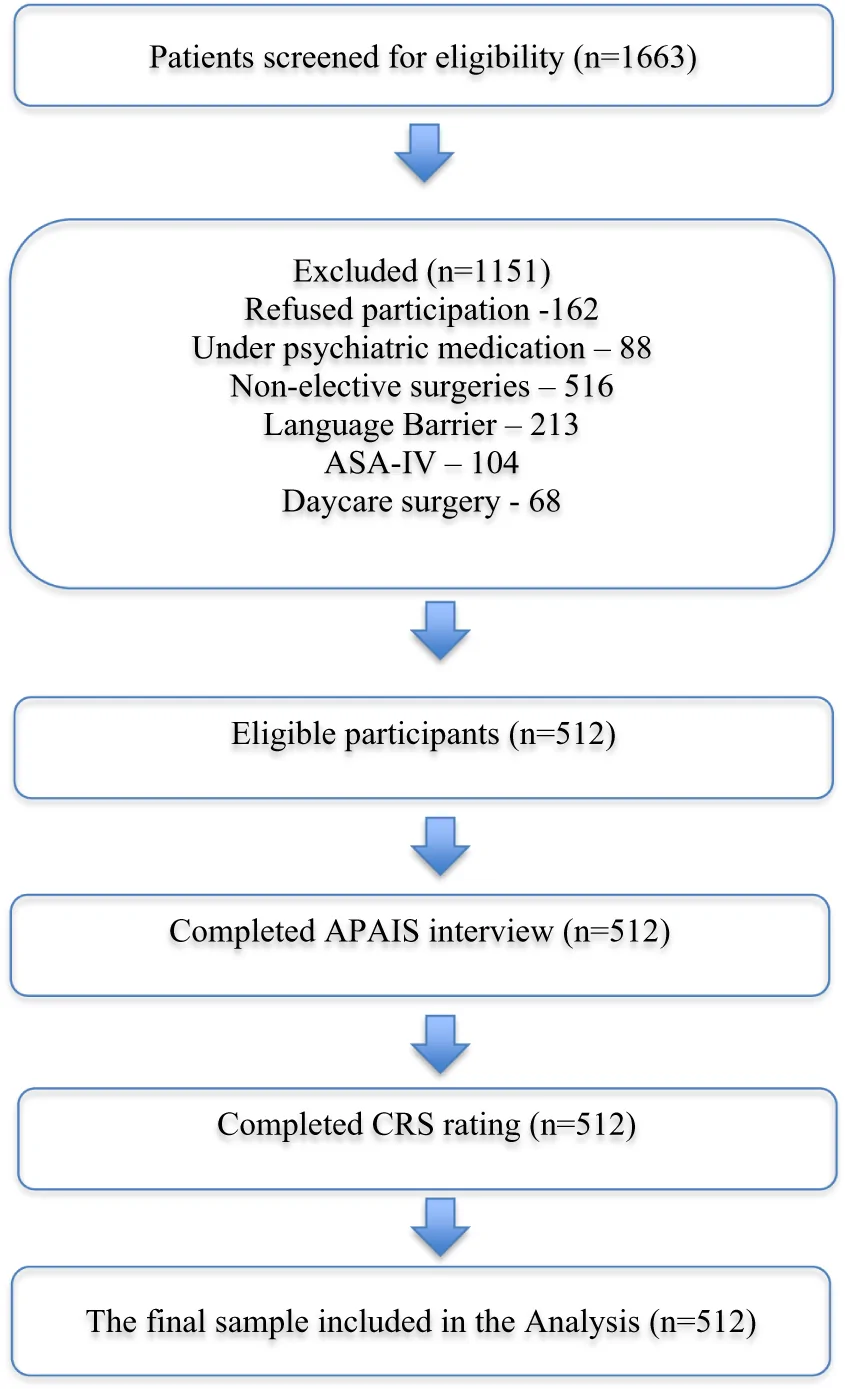
Flow diagram of participants’ screening, exclusion, and final enrolment (*n* = 512).

A total of 1663 patients were screened for eligibility (Fig. 1). Of these, 512 adult patients scheduled for elective surgery met the inclusion criteria and were included in the study. The mean age was 42.2 ± 14.8 years, ranging from 18 to 87 years, with a predominance of female participants (59.8%). Most patients were married (88.5%) and from the Terai region (85.9%). Most were classified as ASA I (66.8%), and nearly three-fourths (73%) had no known comorbidities. More than half of the participants (56.3%) had no previous history of surgery or anesthesia (Table 1).

**Table 1:** Sociodemographic, clinical characteristics, and Bivariate association of high preoperative anxiety (APAIS ≥ 11) with socio-clinical variables of the study participants.

Characteristic	Category	Frequency *n* (%) (*n* = 512)	High anxiety (%) (*n* = 57)	*P*-value
Age group	< 40 years	244 (47.7%)	34 (59.6%)	**0.005**
	40–60 years	196 (38.3%)	18 (31.6%)	
	60 years	72 (14.0%)	5 (8.8%)	
Sex	Male	206 (40.2%)	15 (26.3%)	**0.023**
	Female	306 (59.8%)	42 (73.7%)	
Marital status	Married	453 (88.5%)	51 (89.4%)	**< 0.001**
	Single	47 (9.2%)	3 (5.3%)	
	Divorced/widowed	12 (2.4%)	3 (5.3%)	
Education	No formal education	118 (23.0%)	8 (14.0%)	0.196
	Up to primary	93 (18.2%)	8 (14.0%)	
	Secondary	240 (46.9%)	35 (61.5%)	
	Bachelor’s and above	61 (11.9%)	6 (10.5%)	
Occupation	Unemployed	129 (25.2%)	2 (5.8%)	**0.011**
	Homemaker	190 (37.1%)	31 (54.4%)	
	Self-employed	126 (24.6%)	10 (17.5%)	
	Employed (Private/public)	114 (13.1%)	9 (15.8%)	
Region of residence	Terai (suburban)	440 (85.9%)	48 (84.2%)	0.687
	Hilly (Rural)	72 (14.1%)	9 (15.8%)	
ASA class	I	342 (66.8%)	33 (57.9%)	0.188
	II	139 (27.1%)	18 (31.6%)	
	III	31 (6.1%)	6 (10.5%)	
Comorbidity	Absent	374 (73.0%)	47 (82.5%)	**0.030**
	Present	138 (27.0%)	10 (27.5%)	
Previous surgery/anesthesia	Yes	224 (43.7%)	17 (29.8%)	**0.025**
	No	288 (56.3%)	40 (70.2%)	
Type of surgery	General surgery	231 (45.1%)	21 (36.8%)	**0.001**
	Gynecological	125 (24.4%)	27 (47.4%)	
	Orthopedic	63 (12.3%)	4 (7.0%)	
	ENT	24 (4.7%)	0 (0%)	
	Urological	67 (13.1%)	5 (8.8%)	
	Neuro/dental	2 (0.4%)	0 (0%)	
Surgery site	Public hospital	147 (28.7%)	26 (45.6%)	**0.003**
	Private hospital	365 (71.3%)	31 (54.4%)	
Anesthesia type	Regional	319 (62.3%)	40 (70.2%)	0.193
	General	193 (37.7%)	17 (29.8%)	
Family type	Nuclear	198 (38.6%)	22 (38.6%)	0.523
	Joint	261 (51.0%)	26 (45.6%)	
	Extended	48 (9.4%)	8 (14.0%)	
	Childless	5 (1.0%)	1 (1.8%)	
High information need	Yes (APAIS info ≥ 5)		40 (71.4%)	**< 0.001**
	No		17 (2.2%)	

APAIS, Amsterdam Preoperative Anxiety and Information Scale; ASA, American Society of Anaesthesiologist; ENT, Ear, nose, and throat.

Statistically significant values were highlighted in bold.

Based on the APAIS, 57 patients (11.1%, 95% CI: 8.3–13.9%) had high preoperative anxiety, whereas 73 patients (14.3%, 95% CI: 11.2–17.3%) were classified as highly anxious using the CRS. A high need for information was less common, seen in 35 patients (6.8%, 95% CI: 4.7–9.0%) using APAIS and 40 patients (7.8%, 95% CI: 5.5–10.1%) using CRS (Table 2). The mean APAIS anxiety score was 6.44 ± 2.98, whereas the mean CRS anxiety score was marginally higher at 7.02 ± 3.10. The mean information need scores were 2.63 ± 1.14 for the APAIS and 2.63 ± 1.23 for the CRS scales, respectively.

**Table 2:** Prevalence of high anxiety and information need (*n* = 512).

Variable	Details
APAIS High Anxiety	11.1% (57/512, 95% CI: 8.3–13.9%)
CRS High Anxiety	14.3% (73/512, 95% CI: 11.2–17.3%)
APAIS High Information Need	6.8% (35/512, 95% CI: 4.7–9.0%)
CRS High Information Need	7.8% (40/512, 95% CI: 5.5–10.1%)

Significant associations were observed in the bivariate analysis. High anxiety was more prevalent in females compared to males (*P* = 0.023) and in patients with one or more comorbid conditions (*P* = 0.030). Anxiety was also notably higher in those undergoing gynecological surgeries as compared to general and other surgeries (*P* = 0.001). Patients undergoing surgery at the public hospital had significantly higher anxiety levels than those at the private hospital (*P* = 0.003). Occupational status also showed a significant association (*P* = 0.011), with housewives (54.4%) and employed individuals (17.5%) reporting higher anxiety. Additionally, high preoperative anxiety was observed in 40 of 288 surgery-naïve patients (13.9%) compared with 17 of 224 with previous surgery (7.6%). This difference was statistically significant (*P* = 0.025). However, education level did not show a significant relationship with anxiety (*P* = 0.196). Interestingly, region of residence (*P* = 0.691), family structure (*P* = 0.523), ASA-PS (*P* = 0.188), and type of anesthesia (*P* = 0.193) did not show statistically significant associations with anxiety. A very strong association was observed between high information need and preoperative anxiety (*P* < 0.001), with 71% of patients classified as anxious also reporting high information need, compared to only 2.2% among those with low anxiety (Table 1).

The multivariable logistic regression model showed a good fit to the data (Hosmer–Lemeshow test, *P* = 0.167), with a Nagelkerke *R*^2^ of 0.404, indicating that approximately 40.4% of the variance in high preoperative anxiety was explained by the model. Multivariable logistic regression identified six independent predictors of high preoperative anxiety. Younger age (<40 years) was a strong factor (OR = 1.87, 95% CI: 1.01–3.48, *P* = 0.046), as was female sex (OR = 2.12, 95% CI: 1.12–4.02, *P* = 0.021). Patients undergoing gynecological surgery were more likely to be anxious compared to those undergoing general surgery (OR = 3.10, 95% CI: 1.51–6.38, *P* = 0.002), and being treated at a public hospital increased the likelihood of anxiety more than twofold (OR = 2.08, 95% CI: 1.13–3.83, *P* = 0.019). Similarly, patients with any prior surgical experience had roughly half the odds of high anxiety compared to those without (OR = 0.51, 95% CI: 0.28–0.92, *P* = 0.025). The most significant predictor, however, was high information need, which elevated the likelihood of anxiety nearly 13 times (OR = 12.9, 95% CI: 4.48–37.60, *P* < 0.001) (Table 3). While bivariate analysis originally indicated that occupation (*P* = 0.011) and the prevalence of comorbidities (*P* = 0.030) were strongly linked with high anxiety, these variables did not persist as independent predictors in the multivariable logistic regression model. When controlling for sex and surgical specialty, the effect of occupation was reduced, and comorbidities lost relevance after age was taken into account.

**Table 3:** Multivariable logistic regression analysis of factors associated with high preoperative anxiety (APAIS ≥ 11) (*n* = 512).

Predictor Variable	Adjusted odds ratio (AOR)	95% CI	*P*-value
Female sex	2.12	1.12–4.02	**0.021**
Younger age (< 40 years)	1.87	1.01–3.48	**0.046**
Gynecological surgery	3.10	1.51–6.38	**0.002**
Previous surgery/anesthesia	0.51	0.28-0.92	**0.025**
Public hospital	2.08	1.13–3.83	**0.019**
High information need (APAIS info ≥ 5)	12.90	4.48–37.60	**<0.001**
Education (higher secondary or more)	0.82	0.42–1.58	0.558
Employed status	1.15	0.59–2.25	0.684
Presence of comorbidity	1.31	0.71–2.44	0.388
ASA class II/III	1.44	0.76–2.72	0.264

Statistically significant values were highlighted in bold.

**Figure 2.**
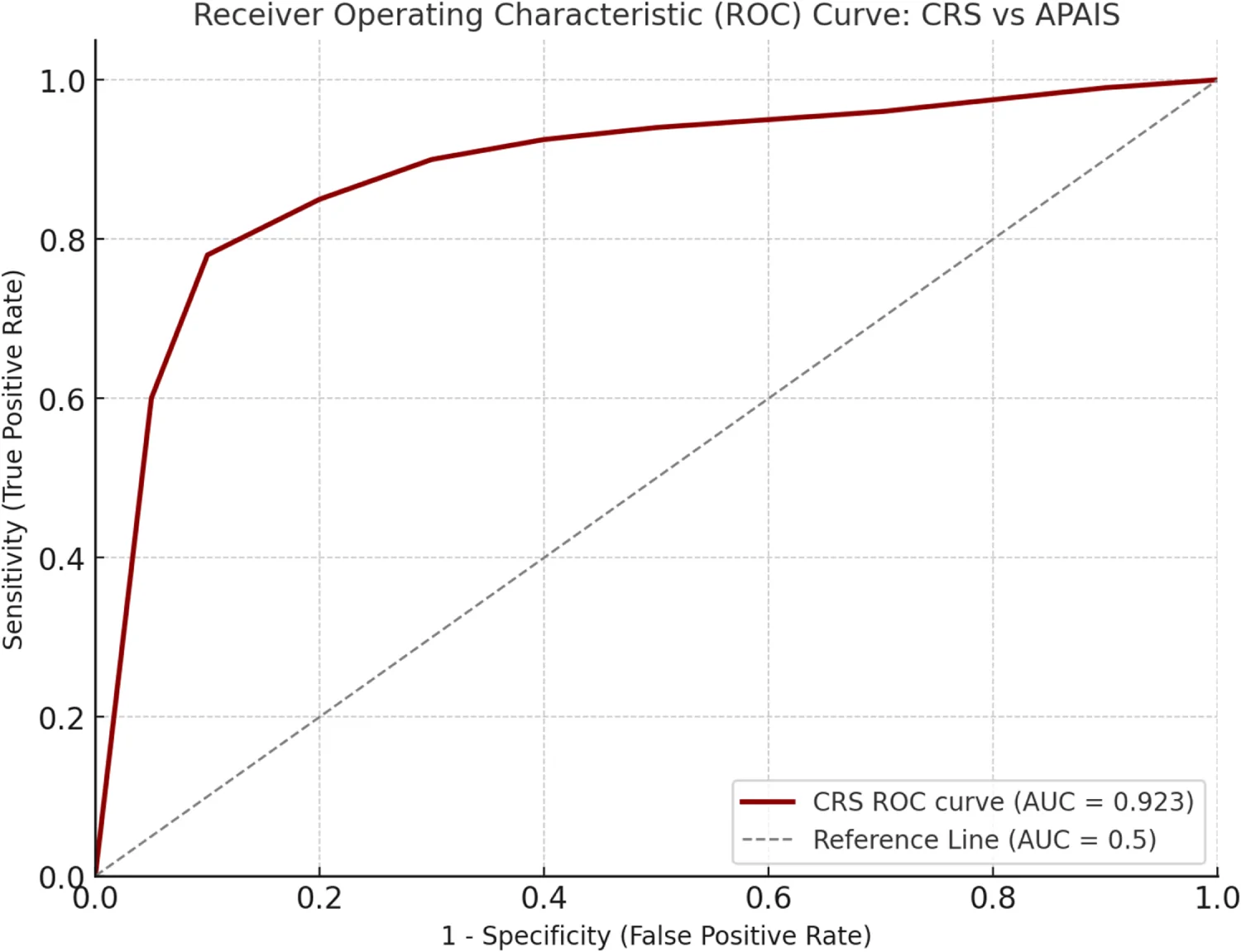
Receiver operating characteristic (ROC) curve illustrating the diagnostic accuracy of the CRS against APAIS-defined anxiety (cutoff ≥11). AUC = 0.923 (95% CI: 0.874–0.972) (*n* = 512). The ROC curve illustrates the diagnostic performance of the CRS in identifying high preoperative anxiety, as defined by an APAIS anxiety score ≥11 (*n* = 512). The *x*-axis represents the false positive rate (1 – specificity), and the *y*-axis represents the true positive rate (sensitivity). The area under the curve (AUC) is approximately 0.923, indicating excellent discriminative ability at a cutoff score >10.

Agreement between APAIS and CRS was substantial, with a Cohen’s kappa of 0.754 and a Phi coefficient of 0.761 (*P* < 0.001), indicating that both tools assessed anxiety in a largely consistent manner. The correlation between the two scales was also strong (Pearson’s *r* = 0.761, 95% CI: 0.722–0.795; Spearman’s *ρ* = 0.761, 95% CI: 0.722–0.795; *P* < 0.001). Internal consistency for both tools was good, with a Cronbach’s *α* of 0.84 for APAIS and 0.81 for CRS. ROC curve analysis demonstrated that CRS performed well in identifying high anxiety, with an area under the curve (AUC) of 0.923 (95% CI: 0.874–0.972; *P* < 0.001) (Fig. 2). At a cutoff of 10.5, CRS showed a sensitivity of 89.5% and specificity of 95.2%, with a Youden’s index of 0.84, confirming its clinical utility in recognizing patient anxiety when self-reports are not feasible.

## Discussion

This study assessed the prevalence and predictors of preoperative anxiety among adults undergoing elective surgeries in Eastern Nepal. Using validated tools such as the APAIS and the CRS, we found that 11.1% of participants experienced high anxiety according to APAIS, and 14.3% according to CRS. This aligns with a previous study by Pokharel *et al*, utilizing the same scale, in Eastern Nepal^[^8^]^. Nonetheless, these findings are markedly lower compared to studies from other regions of Nepal and LMICs globally, where reported rates range from 25.85% to over 83.9% in similar surgical settings, with a pooled prevalence of 55.7%^[^4,6,9–11,17^]^.

The timing of our assessment, 10–15 minutes before transfer to the operating theatre, was chosen to capture the peak of situational anxiety^[^8^]^. Theoretically, anxiety levels are expected to be higher at this stage compared to assessments conducted 24 hours prior in a ward setting, which primarily capture anticipatory anxiety related to the surgical diagnosis. Interestingly, despite this “peak” timing, our observed prevalence was lower than that reported in ward-based studies conducted in similar regions.

Comparatively low anxiety prevalence likely stems from methodological, cultural, and systemic factors. From a systemic and methodological standpoint, each patient at these hospitals underwent a face-to-face pre-anesthetic consultation that addressed worries and recovery expectations. The involvement of anesthesiologists in preoperative counselling, as advocated by Khadka *et al*^[^17^]^, in the hospitals involved and the timing of measurements (10–15 minutes before transfer) seem to have led to patients feeling informed and prepared, potentially contributing to reduced anxiety levels. Eastern Nepal commonly observes sociocultural factors such as strong family support, community cohesion, religious coping mechanisms, and the potential for social desirability bias. These social structures and religious beliefs might promote an attitude of fatalism (“accepting fate”) that reduces overt anxiety^[^18^]^. Furthermore, many patients originate from rural areas and may possess lower health literacy, place trust in physicians’ judgement, and minimize their concerns. Additionally, in Eastern Nepal, cases for elective surgery reflect community trends. The successful operation and recovery of one neighbor motivates other neighbors to attend the hospital. When a neighbor had surgery, their positive personal experiences mitigated their fear. Nevertheless, in the absence of a standardized psychological profile for this demographic, these characteristics remain conjectural. Consequently, qualitative research can investigate the lived experiences and coping strategies of patients in this area.

Age was found to be a significant predictor of anxiety. Participants younger than 40 years were nearly three times more likely to experience high anxiety than their older counterparts. Similar age-related patterns have been reported in multiple international studies^[^6,8,19–21^]^, where younger individuals often show heightened psychological sensitivity to uncertainty, pain, and loss of control associated with surgical procedures. Older patients may draw on previous surgical experiences or life resilience, contributing to lower anxiety levels.

Female sex was another independent risk factor. This finding is consistent with extensive literature indicating that women are generally more susceptible to anxiety disorders and demonstrate heightened emotional responses to stressors, including medical procedures^[^6,20,22–24^]^. Hormonal, sociocultural, and psychological factors, such as differing expressions of fear and help-seeking behavior, may also influence the increased anxiety among females.

Anxiety levels also varied depending on the type of surgery. Patients undergoing gynecological surgeries demonstrated significantly higher anxiety than those undergoing surgeries in other specialties. This may be due to the intimate and reproductive nature of such procedures, fear of complications, or concerns about future fertility and body image. Other Asian and international studies have observed similar findings^[^4,6,19,22,25,26^]^.

Patients undergoing surgery in the public hospital reported significantly higher anxiety compared to those in the private facility^[^27,28^]^. This may indicate variations in the hospital environment, quality of doctor–patient communication, waiting times, or perceived quality of care. In our study, despite the provision of counselling in both hospital settings, patients experienced heightened anxiety levels before surgery in public hospitals due to the extended waiting times. However, we did not collect quantitative data on waiting times, so this interpretation remains speculative. On the other hand, private institutions often offer more personalized attention, shorter surgical waiting times, and better infrastructure, all of which can mitigate psychological stress.

In our study, patients with prior surgical experience reported a 49% reduction in anxiety levels. This finding aligns with previous research suggesting that familiarity with the surgical procedure alleviates psychological stress during subsequent procedures^[^6,9,10,12^]^. Past surgical experience may lead to greater familiarity with the hospital environment, procedural steps, and anticipated outcomes, reducing apprehension about the unknown – a common preoperative stressor.

In our bivariate analysis, housewives reported higher levels of anxiety than employed individuals. However, the significance was lost in the multivariable model. This variation is most likely due to confounding by sex and type of surgery, specifically female and gynecological surgeries, both of which were strong independent predictors of anxiety. Similarly, comorbidities were significant in the bivariate analysis but not in the final regression model. This can be attributed to the confounding influence of age. Once age was factored into the model, the independent effect of comorbidities on preoperative anxiety was no longer statistically significant.

Interestingly, educational status did not show a significant correlation with preoperative anxiety in this study’s multivariate analysis, despite previous studies producing inconsistent findings. Some authors have reported higher anxiety among less-educated patients due to lower health literacy^[^29,30^]^, while others have found greater anxiety among the educated due to increased awareness of potential risks^[^6,8,31^]^. In our population, the majority had secondary or higher education, which may have contributed to more consistent expectations and understanding of the surgical process.

High information needs appeared as the most significant predictor of anxiety. Patients who sought further information regarding their surgery and anesthesia exhibited a significantly increased risk of experiencing worry. This study highlights the critical necessity of excellent communication and patient comprehension in creating psychological preparedness for surgery. Multiple mechanisms may explain this connection. Patients with poor health literacy or previous adverse healthcare experiences may regard surgery as a new danger, thereby raising anxiety about potential complications and anesthesia. Patients may experience unresolved questions due to inadequate communication, which exacerbates their uncertainty and distress. This observation underlines the need for effective preoperative communication, particularly in patients who proactively seek reassurance^[^17^]^. Tailoring preoperative education to meet individual informational requirements may act as a helpful technique to minimize anxiety.

The CRS found a higher proportion of patients with high anxiety (14.3%) than the patient-reported APAIS (11.1%). CRS takes into account both verbal and nonverbal clues, including unrest, tone of voice, and physiological markers. Patients may underreport anxiety due to denial, stoicism, or cultural influences, but clinicians, particularly those with significant expertise, may be more attentive to subtle behavioral clues, leading to higher anxiety ratings when using CRS. CRS had high diagnostic accuracy (AUC = 0.923) and a significant agreement with APAIS (κ = 0.754). In resource-constrained settings, where patient self-report instruments may be impractical due to literacy or time constraints, CRS could serve as a quick and reliable method for assessing anxiety.

This study possesses multiple strengths, such as the use of validated scales, a sufficiently large sample size, and the incorporation of both public and private hospital environments. Nonetheless, limitations must be acknowledged. We recognize that convenience sampling may lead to selection bias. This strategy was used due to logistical challenges; to minimize bias, we consecutively approached every eligible patient during regular clinical hours until the sample size was achieved. The study was restricted to two hospitals in a particular region and did not collect objective data on wait times or other hospital process indicators, perhaps constraining the generalizability of the findings. The omission of emergency, day-care, and pediatric patients may lead to an underestimation of the actual burden of perioperative anxiety. We acknowledge that despite attempts to standardize CRS evaluations via training and supervision, the lack of rigorous inter-rater reliability testing and validation against an independent, objective “gold standard” beyond the self-reported APAIS constitutes a limitation of our work. Furthermore, evaluations were performed at various times throughout the day without rigorous standardization, and variations in anxiety stemming from circadian or environmental influences may have contributed to further score variability.

Despite these limitations, the study highlights the clinical importance of assessing preoperative anxiety and addressing its contributing factors. Early identification and targeted counselling of high-risk patients, particularly younger, surgery-naïve females with high information needs undergoing gynecological surgery in public hospitals, could improve surgical experiences and outcomes. To better understand the long-term impact of preoperative anxiety, future research should look beyond cross-sectional prevalence estimates. Randomized or quasi-experimental trials evaluating structured counselling, decision aids, or tailored information provision could clarify most effective interventions. Expanding qualitative and quantitative research to pediatric and emergency surgical populations would provide a more comprehensive picture of perioperative anxiety in LMIC settings.

## Conclusion

This study found a low prevalence of high preoperative anxiety among adult patients undergoing elective surgery in Eastern Nepal. Despite this, anxiety was much higher among younger people, female patients, those receiving care at a public hospital, and those who had high information needs. These findings highlight the importance of both individual and institutional factors in determining patients’ psychological readiness.

The CRS demonstrated substantial correlation and diagnostic accuracy when compared to the validated self-report APAIS tool, showing its utility in regular clinical practice, particularly in time-constrained settings. Incorporating patient-centered screening and counselling into preoperative protocols may improve perioperative care, particularly for at-risk groups. More multi-center studies in Nepal’s different environments and qualitative studies are necessary to better understand anxiety dynamics and assess the effectiveness of targeted therapies.

## Data Availability

The data that support the findings of this research are available from the corresponding author upon reasonable request.

## Research registration unique identifying number (UIN)

NCT07117578.

## References

[1] Baagil H Baagil H Gerbershagen MU. Preoperative anxiety impact on anaesthetic and analgesic use. Medicina (B Aires) 2023;59:2069. 10.3390/medicina59122069 PMC10744982 38138172

[2] Shebl MA Toraih E Shebl M. Preoperative anxiety and its impact on surgical outcomes: a systematic review and meta-analysis. J Clin Transl Sci 2025;9:e33. 40052059 10.1017/cts.2025.6 PMC11883570

[3] Deffenbacher JL Hazaleus SL. Cognitive, emotional, and physiological components of test anxiety. Cognit Ther Res 1985;9:169–80.

[4] Abate SM Chekol YA Basu B. Global prevalence and determinants of preoperative anxiety among surgical patients: a systematic review and meta-analysis. Int J Surg Open 2020;25:6–16. 10.1016/j.ijso.2020.08.006 PMC7440086 34568611

[5] Bedaso A Bedaso A Mekonnen N. Prevalence and factors associated with preoperative anxiety among patients undergoing surgery in low-income and middle-income countries: a systematic review and meta-analysis. BMJ Open 2022;12:e058187. 10.1136/bmjopen-2021-058187 PMC8919464 35277412

[6] Acharya S Gurung R Parajuli B. Preoperative anxiety assessment in adult patients undergoing elective surgeries: a cross-sectional observational study. J Inst Med (Nepal) 2020;42:18–22.

[7] Moerman N van Dam FSAM Muller MJ. The Amsterdam Preoperative Anxiety and Information Scale (APAIS). Anesth Analg 1996;82:445–51. 8623940 10.1097/00000539-199603000-00002

[8] Pokharel K Bhattarai B Tripathi M. Nepalese patients’ anxiety and concerns before surgery. J Clin Anesth 2011;23:372–78. 21802628 10.1016/j.jclinane.2010.12.011

[9] Dhungana M Limbu R Shrestha M. Assessment of pre-operative anxiety among patients in selected hospitals of Rupandehi, Nepal. J Psychiatr Assoc Nepal 2019;8:28–32.

[10] Lakhe G Shrestha BB Subedi A. Preoperative anxiety among patients undergoing elective surgery in a tertiary care centre: a descriptive cross-sectional study. JNMA J Nepal Med Assoc 2022;60:681–84. 36705210 10.31729/jnma.7636 PMC9446492

[11] Sigdel S Ozaki A Basnet M. Anxiety evaluation in Nepalese adult patients awaiting cardiac surgery. Medicine (Baltimore) 2020;99:e19302. 32118748 10.1097/MD.0000000000019302 PMC7478669

[12] Adhikari SP Pathak BD Ghimire B. Prevalence of pre-operative anxiety and associated risk factors among patients awaiting elective surgery in a tertiary care hospital. F1000Res 2023;12:1207. 38318155 10.12688/f1000research.136320.2 PMC10839854

[13] Mavridou P Dimitriou V Manataki A. Patient’s anxiety and fear of anesthesia: effect of gender, age, education, and previous experience of anesthesia. A survey of 400 patients. J Anesth 2013;27:104–08. 22864564 10.1007/s00540-012-1460-0

[14] Caumo W Schmidt AP Schneider CN. Risk factors for preoperative anxiety in adults. Acta Anaesthesiol Scand 2001;45:298–307. 11207465 10.1034/j.1399-6576.2001.045003298.x

[15] Agha RA Mathew G Rashid R. Revised strengthening the reporting of cohort, cross-sectional and case-control studies in surgery (STROCSS) guideline: An update for the age of Artificial Intelligence. Premier J Sci 2025;10:100081.

[16] Agha RA Mathew G Rashid R. Transparency in the reporting of artificial intelligence – the TITAN guideline. Prem J Sci 2025;10:100082.

[17] Khadka B Sharma A Bhattarai PR. Role of preoperative counselling with NSQIP surgical risk calculator in the surgical patients. Surg Open Sci 2024;18:11–16. 38312306 10.1016/j.sopen.2024.01.007 PMC10831096

[18] Celik F Edipoglu IS. Evaluation of preoperative anxiety and fear of anesthesia using APAIS score. Eur J Med Res 2018;23:41. 30205837 10.1186/s40001-018-0339-4 PMC6131845

[19] Friedrich S Reis S Meybohm P. Preoperative anxiety. Curr Opin Anaesthesiol 2022;35:674–78. 36131642 10.1097/ACO.0000000000001186

[20] Yu J Zhang Y Yu T. Preoperative anxiety in chinese adult patients undergoing elective surgeries: a multicenter cross-sectional study. World J Surg 2022;46:2927–38. 36070012 10.1007/s00268-022-06720-9 PMC9636076

[21] Eberhart H Aust M Schuster T. Preoperative anxiety in adults: a cross-sectional study on specific fears and risk factors. BMC Psychiatry 2020;20:140. 32228525 10.1186/s12888-020-02552-w PMC7106568

[22] Dibabu AM Ketema TG Beyene MM. Preoperative anxiety and associated factors among women admitted for elective obstetric and gynecologic surgery in public hospitals, Southern Ethiopia: a cross-sectional study. BMC Psychiatry 2023;23:728. 37807071 10.1186/s12888-023-05005-2 PMC10561508

[23] Mulugeta H Ayana M Sintayehu M. Preoperative anxiety and associated factors among adult surgical patients in debre markos and felege hiwot referral hospitals. Northwest Ethiopia, BMC Anesthesiol 2018;18:155. 30376809 10.1186/s12871-018-0619-0 PMC6208029

[24] Bagle A Yerramshetty M Garud IG. Comparison between effect of preoperative verbal counseling versus preoperative counseling using anesthesia information sheet on anxiety of patients undergoing elective surgeries: a randomized comparative study. Cureus 2024. doi:10.7759/cureus.64667 PMC11326750 39149646

[25] Bansal T Joon A. Preoperative anxiety—an important but neglected issue: a narrative review. Indian Anaesth Forum 2016;17:37.

[26] Laufenberg-feldmann R Kappis B. Assessing preoperative anxiety using a questionnaire and clinical rating: a prospective observational study. Eur J Anaesthesiol 2013;30:758–63. 23787971 10.1097/EJA.0b013e3283631751

[27] Shawahna R Jaber M Maqboul I. Prevalence of preoperative anxiety among hospitalized patients in a developing country: a study of associated factors. Perioper Med 2023;12:47. 10.1186/s13741-023-00336-w PMC10463373 37620871

[28] Sigdel S. Perioperative anxiety: a short review. Glob Anesth Perioper Med 2015;1. doi:10.15761/GAPM.1000126

[29] Nuri A Abute L Elilo LT. Assessment of preoperative anxiety levels among patients admitted for surgery in public hospitals, Southern Ethiopia. SAGE Open Nurs 2024;10:23779608241274191. 39185502 10.1177/23779608241274191 PMC11342311

[30] Ossai EN Nwosu ADG Onwuasoigwe O. Prevalence and predictors of anxiety among surgical patients in the preoperative holding area of national orthopaedic hospital, Enugu, Nigeria: a cross-sectional study. J West Afr Coll Surg 2023;13:105–12. 37228877 10.4103/jwas.jwas_10_23 PMC10204921

[31] Khalili N Karvandian K Eftekhar Ardebili H. Predictors of preoperative anxiety among surgical patients in Iran: an observational study. Aacc 2019;6:2044.

